# ATG7 is dispensable for LC3–PE conjugation in thioglycolate-elicited mouse peritoneal macrophages

**DOI:** 10.1080/15548627.2021.1874132

**Published:** 2021-01-18

**Authors:** Nemanja Vujić, Ivan Bradić, Madeleine Goeritzer, Katharina B. Kuentzel, Silvia Rainer, Dagmar Kratky, Branislav Radović

**Affiliations:** a Gottfried Schatz Research Center, Medical University of Graz, Graz, Austria; b BioTechMed-Graz, Graz, Austria

**Keywords:** ATG3, ATG5, ATG7, LC3 lipidation, LC3-II, macrophages

## Abstract

Thioglycolate-elicited macrophages exhibit abundant conjugation of LC3 with PE (LC3-II). Among other autophagy-related (ATG) proteins, it is proposed that, like in yeast, both ATG5 and ATG7 are essential for LC3 conjugation. Using *atg5*-deficient (*^−/-^*) and *atg7^−/-^*macrophages, we provide evidence that loss of ATG5 but not of ATG7 resulted in LC3-II depletion. Accumulation of LC3-II in elicited *atg7^−/-^* macrophages in response to bafilomycin A_1_ validated these data. Furthermore, complete loss of ATG3 in *atg7*^−/-^ macrophages demonstrated that ATG7 and ATG3 are dispensable for LC3–PE conjugation. In contrast to thioglycolate-elicited macrophages, naïve peritoneal and bone marrow-derived *atg7^−/-^* macrophages exhibited no LC3-II, even under inflammatory stimuli *in vitro*. Hence, the macrophage metabolic status dictates the level of LC3–PE conjugation with a supportive but nonessential role of ATG7, disclosing the eukaryotic exception from the LC3 lipidation model based on yeast data. **Abbreviations**: ATG: autophagy-related; BM: bone marrow; MAP1LC3/LC3: microtubule-associated protein 1 light chain 3; PE: phosphatidylethanolamine.

## Introduction

MAP1LC3/LC3 (microtubule-associated protein 1 light chain 3) is involved in diverse cellular processes linked to sequestration, transport, and degradation of cellular material such as macroautophagy/autophagy [1], phagocytosis [2], and endocytosis [3]. In all these processes, active LC3 is generated by covalent binding (lipidation) with phosphatidylethanolamine (PE). LC3 is one of the mammalian homologs of yeast Atg8 [4], whose lipidation requires many autophagy-related (Atg) proteins involved in *de novo* formation of a double-membrane vesicle. Especially Atg5 (substrate), Atg7, Atg3, and Atg10 (enzymes) are critical for ligation with PE [5]. Similar to ubiquitination, Atg12 is first conjugated to Atg5 by Atg7 (E1-like-) and Atg10 (E2-like enzyme). The Atg12–Atg5 complex (E3-like) facilitates conjugation of PE to Atg8 by Atg7 and its corresponding E2-like enzyme Atg3, supporting expansion of phagophore membranes [6]. Out of several mammalian homologs of yeast Atg8, LC3 is the best characterized. It is proposed that LC3 undergoes a modification process similar to Atg8 [4,7]. ATG7 and ATG3 catalyze transformation of cytosolic LC3-I to a membrane-bound form (LC3-II), which corresponds to Atg8–PE in yeast [6].

We investigated the consequences of ATG5 and ATG7 deficiency on LC3-II formation in murine macrophages which utilize LC3-II for canonical and/or non-canonical function(s) [2] and found diverging results between thioglycolate-elicited and non-elicited cells.

## Results and discussion

### Loss of ATG5 but not ATG7 depletes LC3-II in thioglycolate-elicited macrophages

**Figure 1.**
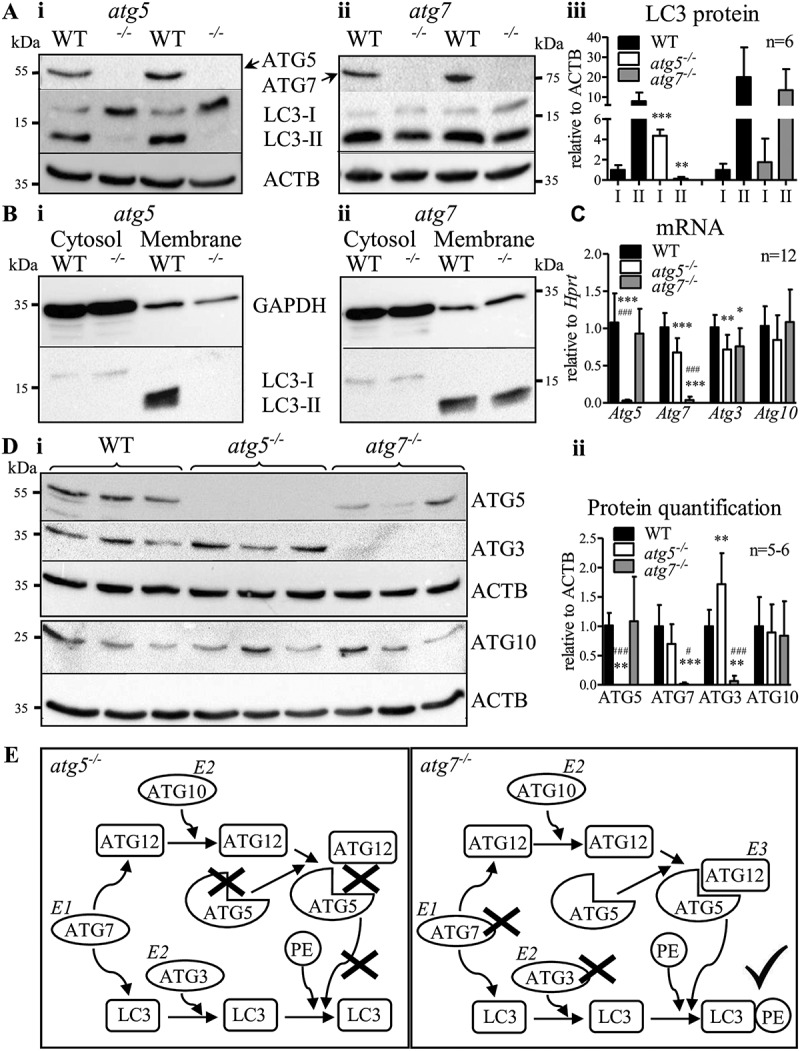
Abolished ATG3 protein expression but comparable LC3–PE lipidation in thioglycolate-elicited *atg7^−/-^* macrophages. Representative immunoblotting of LC3, ATG5, ATG7, and ß-actin in whole-cell lysates isolated from thioglycolate-elicited WT, (**Ai**) *atg5^−/-^*, and (**Aii**) *atg7^−/-^* peritoneal macrophages. Each lane represents a macrophage sample from an individual mouse. (**Aiii**) Quantification of LC3-I and LC3-II in *atg5^−/-^, atg7^−/-^*, and corresponding control macrophages normalized to ACTB/β-actin (n = 6). Statistics were calculated using Student’s t-test (two-tailed) with Welch correction for unequal variances; ** p < 0.01, *** p < 0.001. LC3 protein abundance in cellular fractions of WT, (**Bi**) *atg5^−/-^*, and (**Bii**) *atg7^−/-^* thioglycolate-elicited peritoneal macrophages with GAPDH as loading control. Each lane represents a sample pool from three mice. (**C**) mRNA expression of *Atg5, Atg7, Atg3*, and *Atg10* in WT, *atg5^−/-^*, and *atg7^−/-^* macrophages relative to *Hprt* (n = 12). (**Ci**) Representative immunoblotting of (ATG12**–**)ATG5, ATG3 (upper blot), and ATG10 (lower blot), using the same samples obtained from three individual mice. (**Dii**) Protein quantification relative to ACTB/β-actin (n = 5–6). Data represent mean values + SD. Statistics in (**C** and **Dii**) were calculated using one-way ANOVA with Bonferroni correction; * relative to WT; ^#^ *atg5^−/-^* relative to *atg7^−/-^*; *^, #^ p < 0.05, ** p < 0.01, ***^/###^ p < 0.001. (E) Schematic presentation of the summarized results

Loss of ATG5 or ATG7 protein (Figure 1Ai,Aii,Di,Dii) and their respective mRNAs (Figure 1C) in macrophages proves the successful generation of the knockout mice. As expected, deletion of *Atg5* resulted in substantial expression of LC3-I and absence of LC3-II (Figure 1Ai, Fig. S1A). However, lack of ATG7 in thioglycolate-elicited macrophages failed to inhibit LC3 lipidation with only minor reduction in LC3-II compared to WT cells (Figure 1Aii, Fig. S1B,C). LC3-II was also found in thioglycolate-elicited *atg7^−/-^* macrophages 2 h after isolation, indicating that lipidation was not induced during culturing of the cells (Fig. S1C). After separation of cytosolic and membrane fractions, LC3-II was absent in the membrane fraction of thioglycolate-elicited *atg5^−/-^* macrophages (Figure 1Bi), whereas the amount of LC3-II in *atg7^−/-^* macrophages was comparable to WT cells. This is an unexpected finding, considering that LC3 lipidation is generally accepted as being dependent on both ATG5 and ATG7 [6].

*Atg10* mRNA (Figure 1C) and ATG10 protein expression (Figure 1D) were comparable between all genotypes. However, ATG3 protein was depleted in *atg7^−/-^* macrophages (Figure 1D) but only reduced by 25% at mRNA level (Figure 1C), indicating posttranscriptional regulation of ATG3. Despite reduced *Atg3* mRNA (Figure 1C), we observed significantly more ATG3 protein in *atg5^−/-^* compared to control macrophages (Figure 1Dii). Whether ATG5 protein hinders the translation of *Atg3* or whether ATG3, as an E2-like enzyme, increases its activity to compensate for the lack of ATG5 requires further investigation. However, ATG3 depletion in *atg7^−/-^* macrophages indicated that the E2-like enzyme ATG3 is redundant without the E1-like enzyme ATG7, similar to *Atg7*- and *Atg3*-independent autophagy in *Drosophila* [8].

We conclude that the E2-like enzyme ATG10 does not require ATG7 for ATG12 activation and conjugation to ATG5 (Figure 1E). Subsequent conjugation of PE to LC3 is possible in the absence of ATG7 and ATG3. Thus, the E3-like ATG12–ATG5 complex is sufficient for conjugation of PE to LC3 in murine thioglycolate-elicited macrophages.

### Lipidated LC3 accumulates in thioglycolate-elicited peritoneal atg7^−/-^ macrophages in response to bafilomycin A_1_

**Figure 2.**
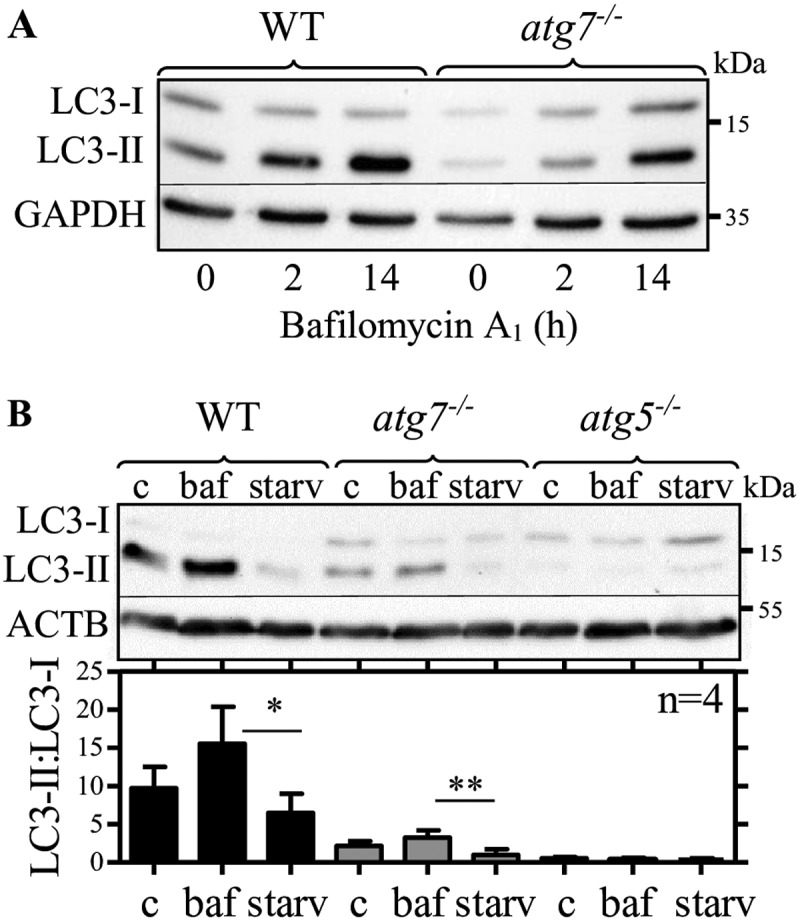
Lipidated LC3 accumulates in thioglycolate-elicited peritoneal *atg7^−/-^* macrophages in response to bafilomycin A_1_. Representative LC3 immunoblotting in WT and *atg7^−/-^* macrophages. (**A**) GAPDH and (**B**) ACTB/β-actin were used as loading controls. Cells were treated for (**A**) 2 h and (**A** and **B**) 14 h with bafilomycin A_1_ (baf), (**B**) starved for 1 h in PBS (starv) or left untreated (**C**). Bars represent mean LC3-II:LC3-I ratios (+ SD) obtained from four individual mice. One-way ANOVA with Bonferroni correction were used to calculate differences between the different treatments; * p < 0.05, ** p < 0.01

To investigate LC3 lipidation dynamics in thioglycolate-elicited peritoneal WT and *atg7^−/-^* macrophages in more detail, we starved (PBS) the cells to induce autophagy and used bafilomycin A_1_ to inhibit autolysosomal LC3 degradation. We observed time-dependent accumulation of LC3-II in elicited WT and *atg7^−/-^* macrophages (Figure 2A), reduction by starvation in both genotypes (Figure 2B), and no induction of LC3-II in *atg5^−/-^* macrophages (Figure 2B). To overcome individual differences in LC3 expression between independent experiments, we used LC3-II:LC3-I ratios instead of normalization to a housekeeping protein. Elicited WT and *atg7^−/-^* macrophages responded to inhibition and stimulation with comparable increase (1.6- versus 1.5-fold) and decrease (44% versus 66%) of LC3-II:LC3-I ratios (Figure 2B). Thus, we conclude that despite reduced LC3 lipidation capacity in elicited *atg7^−/-^* macrophages LC3 lipidation dynamics are functional in response to autophagy inhibition and stimulation, respectively.

### LC3-II is absent in non-elicited peritoneal and bone marrow-derived atg7^−/-^ macrophages

**Figure 3.**
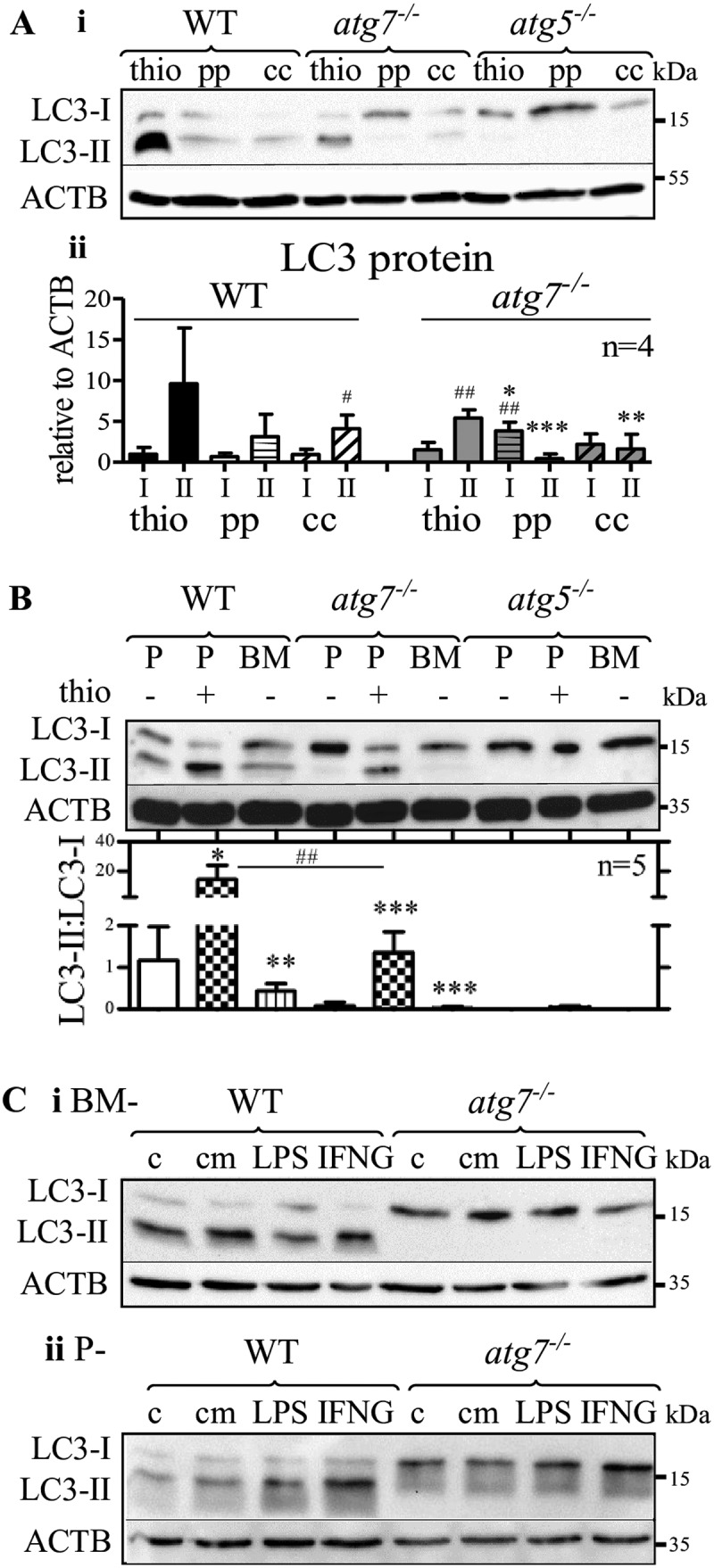
Non-elicited peritoneal and BM-derived *atg7^−/-^* macrophages do not conjugate PE to LC3. (**A**) Comparison of LC3 protein expression in WT, *atg7^−/-^*, and *atg5^−/-^* peritoneal macrophages elicited by thioglycolate (thio), proteose-peptone (pp), and concanavalin A (cc). (**Ai**) Representative LC3 immunoblot with ACTB/β-actin as a loading control. (**Aii**) Mean LC3 expression values (+ SD) obtained from four individual mice. One-way ANOVA with Bonferroni correction were used to calculate differences between the different treatments; * p < 0.05, ** p < 0.01, *** p < 0.001. Student’s t-test (two-tailed) with Welch correction was used to compare LC3-II with LC3-I; ^#^ p < 0.05, ^##^ p < 0.01. (**B**) Representative LC3 immunoblotting in non-elicited peritoneal (P-), thioglycolate (thio)-elicited (P+), and BM-derived (BM-) WT, *atg5^−/-^*, and *atg7^−/-^* macrophages, respectively. Bars represent mean LC3-II:LC3-I ratios + SD (n = 5). Statistics were calculated using one-way ANOVA with Bonferroni correction. * differences by treatment; ^#^ differences by genotype; * p < 0.05, **^/##^ p < 0.01, *** p < 0.001. LC3 immunoblotting in (**Ci**) BM- and (**Cii**) P- WT and *atg7^−/-^* macrophages treated with lipopolysaccharide (LPS), IFNG (interferon gamma), thioglycolate (thio), conditioned media (cm) collected after incubation of thioglycolate-elicited WT macrophages for 24 h, or left untreated (**C**). ACTB/β-actin was used as loading control

To examine whether other inflammatory agents stimulate LC3 lipidation in *atg7^−/-^* macrophages *in vivo*, we compared LC3 protein expression in peritoneal macrophages elicited by thioglycolate, proteose-peptone, and concanavalin A (Figure 3A). LC3-II was hardly detectable in proteose-peptone elicited cells but we observed LC3-II in *atg7^−/-^* macrophages elicited by concanavalin A (Figure 3Ai). However, if compared with LC3-II from thioglycolate-elicited *atg7^−/-^* cells, LC3-II expression was significantly reduced in concanavalin A-elicited macrophages (Figure 3Aii). Moreover, only thioglycolate-elicited *atg7^−/-^* macrophages exhibited significantly higher LC3-II than LC3-I expression, in contrast to WT macrophages showing increased LC3-II by all eliciting agents. We conclude that among investigated inflammatory stimuli peritoneal injection of thioglycolate induces LC3 lipidation in *atg7^−/-^* macrophages most effectively. However, low expression of LC3-II in cells isolated after concanavalin A injection may be a result of a reduced macrophage response, considering that thioglycolate is more potent than other eliciting agents to induce peritonitis and yield a greater number of cells in the peritoneum [9]. These results indicated that pronounced inflammation is necessary to boost LC3 lipidation in *atg7^−/-^* macrophages.

In line with these results, non-elicited peritoneal and bone marrow (BM)-derived *atg7^−/-^* macrophages were unable to conjugate PE to LC3 (Figure 3B). As expected, LC3-II was absent in all types of *atg5^−/-^* macrophages. If compared with non-elicited peritoneal and BM-derived macrophages, the LC3-II:LC3-I ratios were drastically higher in thioglycolate-elicited WT and *atg7^−/-^* macrophages. Consistent with data in Figure 2B, the LC3-II:LC3-I ratio was markedly reduced in elicited *atg7^−/-^* compared with WT macrophages.

The induction of an inflammatory response by thioglycolate [10] is accompanied by increased macrophage phagocytosis and lysosomal activity [11]. Besides, granulocyte-macrophage colony-stimulating factor induces LC3 lipidation in human monocytes [12]. We therefore investigated whether pro-inflammatory stimuli [13,14] trigger the lipidation of LC3 in *atg7^−/-^* macrophages *in vitro*. BM-derived (Figure 3Ci) and non-elicited peritoneal (Figure 3Cii) *atg7^−/-^* macrophages showed only non-lipidated LC3-I after incubation with lipopolysaccharide and interferon gamma. Moreover, LC3-II bands were absent in *atg7^−/-^* macrophages after *in vitro* treatment with conditioned medium from thioglycolate-elicited WT macrophages (Figure 3C) or with thioglycolate *in vitro* [10] (Fig. S2).

It is likely that inflammatory responses to thioglycolate trigger the LC3 lipidation in nonresident *atg7^−/-^* macrophages differentiated from recruited monocytes. Though additional experiments are required to understand the mechanism of LC3-lipidation in *atg7^−/-^* macrophages after i.p. injection of thioglycolate in *atg7^−/-^* mice, this process likely occurs only *in vivo*. It remains unclear whether the peritoneal architecture and the interaction with the surrounding tissues and the extracellular matrix or the presence of other cells in the peritoneal cavity are required. Numerous myeloid and lymphoid cells found in the peritoneal inflammatory exudate [15] might be involved. Neutrophils, which are plentiful in the peritoneum 24 h after thioglycolate injection [10] and 6 h after administration of concanavalin A [16], are the most interesting cells for future investigation.

We conclude that the macrophage activation status dictates the level of LC3–PE conjugation. In contrast to ATG5, the role of ATG7 and ATG3 in this process is supportive but not essential.

## Materials and methods

### Reagents

The following reagents were used: thioglycolate broth containing (per liter of deionized water) 17 g peptone from casein (1654) and 3 g peptone from soy (1657) from GERBU Biotechnik GmbH, 6 g glucose (X997), 2.5 g NaCl (3957), 0.1 g Na_2_SO_3_ (P033) from Carl Roth, 0.5 g sodium thioglycolate (T0632) and 0.7 g agar (A1296) from Sigma-Aldrich; RIPA buffer containing 150 mM NaCl, 1% Triton® X-100 (3051), 0.1% SDS (CN30), 50 mM Tris, pH 8 (9140), 1 mM DTT (6908) from Carl Roth, 0.5% Na-deoxycholate (Merck, 1065040250), and protease inhibitor cocktail (Sigma-Aldrich, P8340); fractionation lysis buffer containing 20 mM HEPES (HN77), 10 mM KCl (HN02), 2 mM MgCl_2_ (A537) and 1 mM DTT (6908) from Carl Roth, 1 mM Invitrogen™ UltraPure™ EDTA, pH 8.0 (Thermo Fisher Scientific, 15575020), 1 mM EGTA (GERBU Biotechnik GmbH, 1310) and protease inhibitor cocktail; Gibco™Bacto™ proteose peptone (Thermo Fisher Scientific, 211684); bafilomycin A_1_ (B1793), concanavalin A (C5275), DMEM (41966029), IFNG/interferon-gamma (SRP3058), lipopolysaccharide (L4391), PBS, pH 7.4 (10010), and penicillin-streptomycin (P4333) from Sigma-Aldrich.

### Animals and cells

Mice with a targeted deletion of *Atg5* or *Atg7* in myeloid cells were generated by crossing [17] *Lyz2/LysM-Cre* with *Atg5^flox/flox^* mice (RIKEN BRC) or *Atg7^flox/flox^* mice [18] (M. Komatsu). Mice were kept in a clean environment with unlimited access to chow diet (Altromin Spezialfutter, 1324) and water. All experiments were approved by the Austrian Federal Ministry of Education, Science, and Research (BMWFW-66010/0076-WF/II/3b/2014, BMWFW-66010/0153-WF/V/3b/2015 and BMBWF-66.010/0023-V/3b/2018).

Peritoneal macrophages were obtained 3 days after intraperitoneal injection of 2 ml of 3% thioglycolate broth, 2 ml of 3% proteose peptone or 25 µg/ml of concanavalin A. Macrophages were isolated by peritoneal lavage with 10 ml ice-cold PBS/EDTA and cultured in high glucose (25 mM) DMEM supplemented with 10% LPDS (Sigma-Aldrich, S5394), 1% penicillin-streptomycin for 2 h to adhere to the non-treated culture dish. Thereafter, cells were washed twice with PBS and cultured in DMEM for 48 h prior to further use. For the bafilomycin A_1_ treatment, cells were incubated as described before for 46- or 34 h and then co-incubated with 10 nM bafilomycin A_1_ for 2 or 14 h, respectively. PBS-starved cells were incubated as described before for 47 h and then incubated in PBS for 1 h. Cells were harvested simultaneously with untreated cells. BM-derived cells were differentiated to macrophages for 7 days in DMEM containing 10% of conditioned medium from L929 cells (ECACC, 85011425). Cells were lysed in RIPA buffer in the presence of 1 mM DTT and protease inhibitor cocktail. For subcellular fractionation, cells were lysed in fractionation lysis buffer by triturating cells through a 27 G needle with visual inspection of successful lysis under the microscope. After separation of the nuclear fraction (2,655 x g), the membrane fraction was obtained by centrifugation at 100,000 x g, while the cytosolic fraction was collected as supernatant. For *in vitro* treatments of naïve peritoneal and BM-derived macrophages, 10 nM bafilomycin A_1_, 100 ng/ml lipopolysaccharide, 5 ng/ml interferon-gamma or 0.3% thioglycolate broth were used. Conditioned media were collected from thioglycolate-elicited WT macrophages after culturing for 24 h in DMEM.

### Western blotting

Protein samples were separated by 15% SDS-polyacrylamide gel electrophoresis transferred to PVDF membranes and incubated with rabbit polyclonal antibodies against ATG3 (Thermo Fisher Scientific, PA5-17018), ATG10 (Abclonal, A7390), ATG5, ATG7, LC3B (Cell Signaling Technology, 4445), GAPDH/glyceraldehyde 3-phosphate dehydrogenase (Cell Signaling Technology, 2118), and mouse monoclonal ACTB/β-actin (Sigma-Aldrich, A2228). HRP-conjugated rabbit anti-mouse (Dako, P0260) and goat anti-rabbit (Thermo Fisher Scientific, 31460) were visualized with ECL (BioRad, 1705061) using the ChemiDoc™ Imaging System (Bio-Rad Laboratories, Hercules, CA).

### Real-time PCR

RNA was isolated using TriFast™ (VWR, 30–2010) and reverse transcribed using the Applied Biosystems^TM^ High Capacity cDNA Reverse Transcription Kit (Thermo Fisher Scientific, 4368813). qPCR was performed on a Bio-Rad CF X96 (Bio-Rad Laboratories, Hercules, CA) with GoTaq® qPCR Mastermix (Promega, A6002), normalized to *Hprt* (hypoxanthine guanine phosphoribosyl transferase) and calculated using the 2^−ΔΔCT^ method. Primer sequences are available upon request.

## Supplementary Material

Supplemental Material

## Data Availability

All data are available from the corresponding author branislav.radovic@medunigraz.at upon reasonable request.
