# Supplementary material for: ATG7 is dispensable for LC3–PE conjugation in thioglycolate-elicited mouse peritoneal macrophages
Source: Autophagy. 2021 Jan 18;17(11):3402–7. doi: 10.1080/15548627.2021.1874132 (PMC8632316; doi:10.1080/15548627.2021.1874132)
Supplement: Supplemental Material [file KAUP_A_1874132_SM9529.docx]

Suplemental material to:

**ATG7 is Dispensable for LC3–PE Conjugation in Thioglycolate-Elicited Mouse Peritoneal Macrophages**

Nemanja Vujić^a,b^, Ivan Bradić^a,b^, Madeleine Goeritzer^a,b^, Katharina B. Kuentzel^a^, Silvia Rainer^a^, Dagmar Kratky^a,b^ and Branislav Radović^a,b,*^

^a^Gottfried Schatz Research Center, Medical University of Graz, Austria. ^b^BioTechMed-Graz, Austria.

**FIGURE S1**

LC3-I

LC3-II

WT ^-/-^ WT ^-/-^ WT ^-/-^ WT ^-/-^


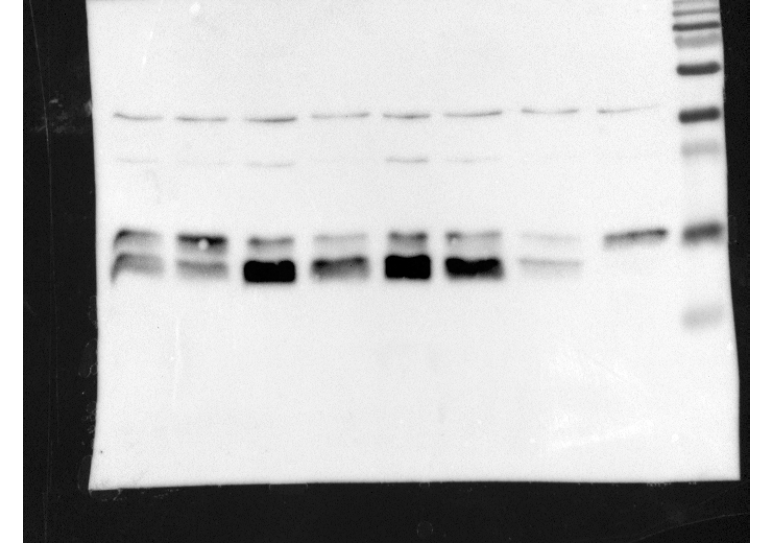

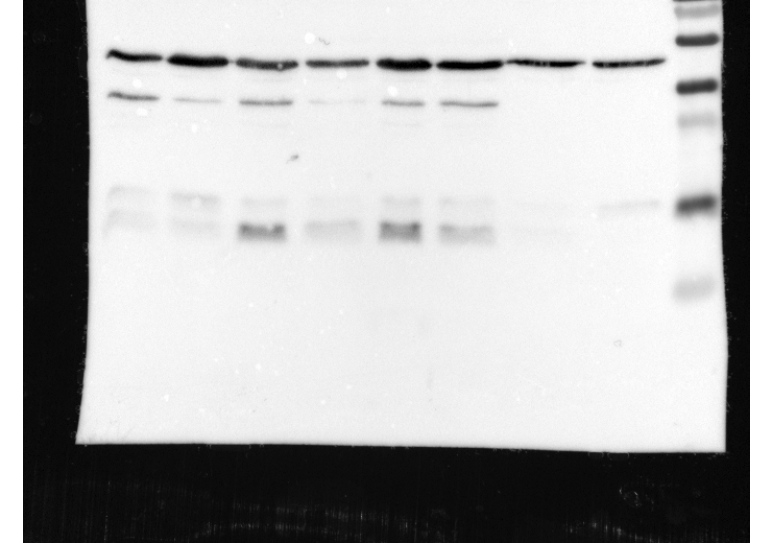


ACTB

10

15

35

55

thioglycolate-elicited non-elicited

**C**

WT vs. *atg7^-/-^*

55

LC3-I

LC3-II

ACTB

WT vs. *atg5^-/-^*

WT ^-/-^ WT ^-/-^ WT ^-/-^ WT ^-/-^

1 2 1 2 1 2


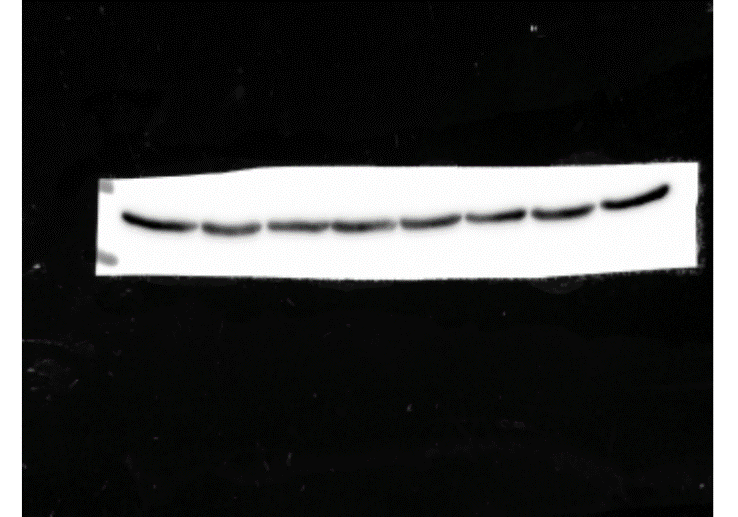


WT ^-/-^ WT ^-/-^ WT ^-/-^ WT ^-/-^


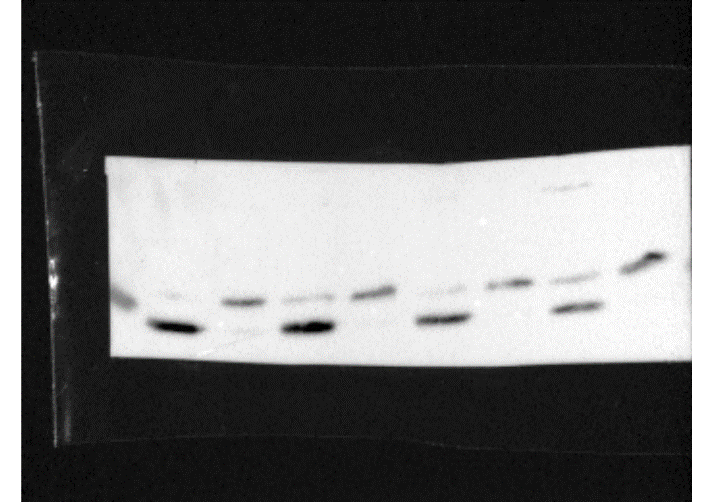

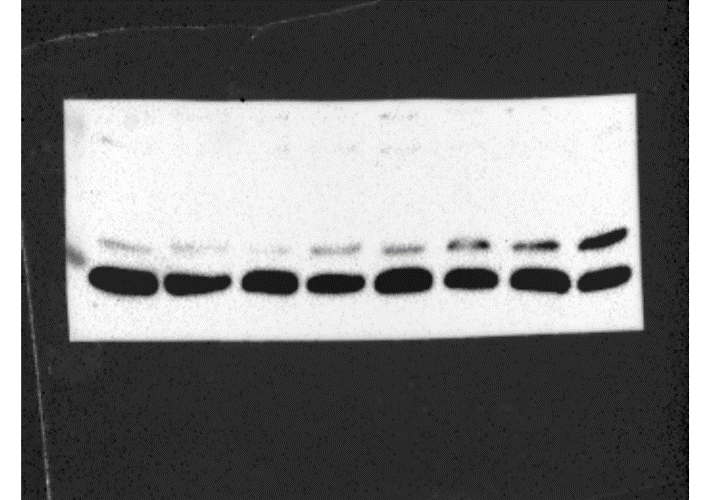

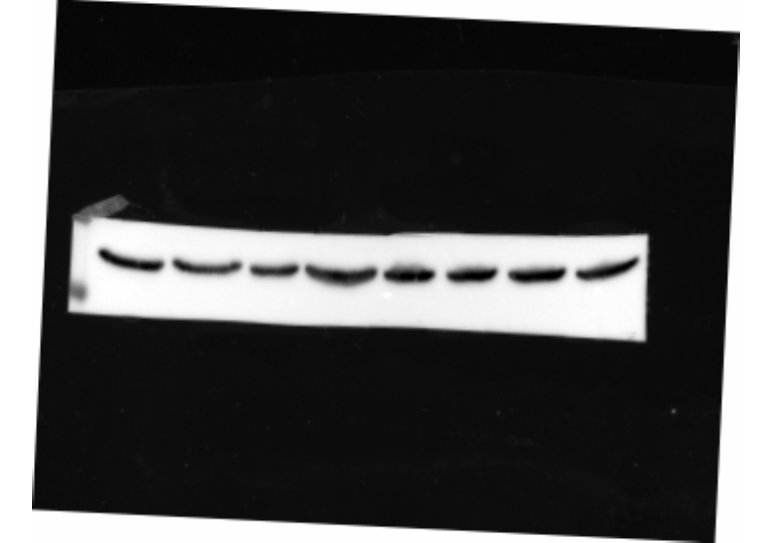


WT vs. *atg7^-/-^*

**A**

**B**

15

kDa D

35

35

15

kDa D

kDa D

**Figure S1**. Substantial LC3-II protein expression in *atg7^-/-^* macrophages. Immunoblotting analysis of the autophagy protein LC3, and ACTB/β-actin (as a loading control) in whole cell lysates isolated from thiglycolate-elicited WT, and (**A**) *atg5^-/-^* and (**B** and **C**) *atg7^-/-^* peritoneal macrophages, respectively. Cells were kept in culture for (**A** and **B**) 48 h or (**C**) only 2 h to remove non-adherent cells.

**FIGURE S2**

LC3-I

LC3-II

ACTB

c thio cm c thio cm kDa

WT *atg7^-/-^*


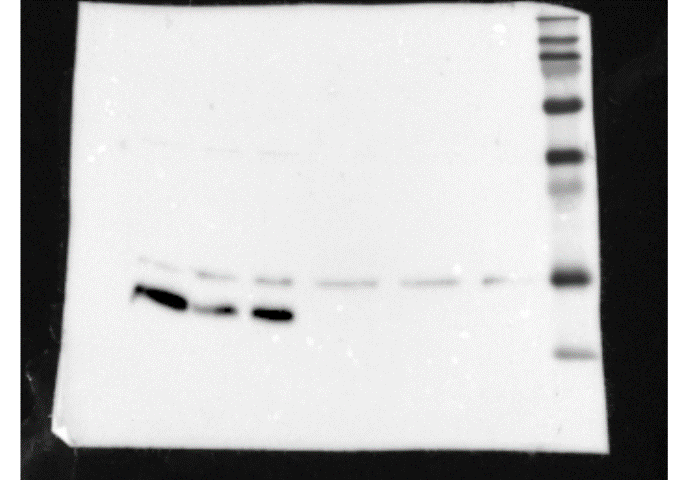

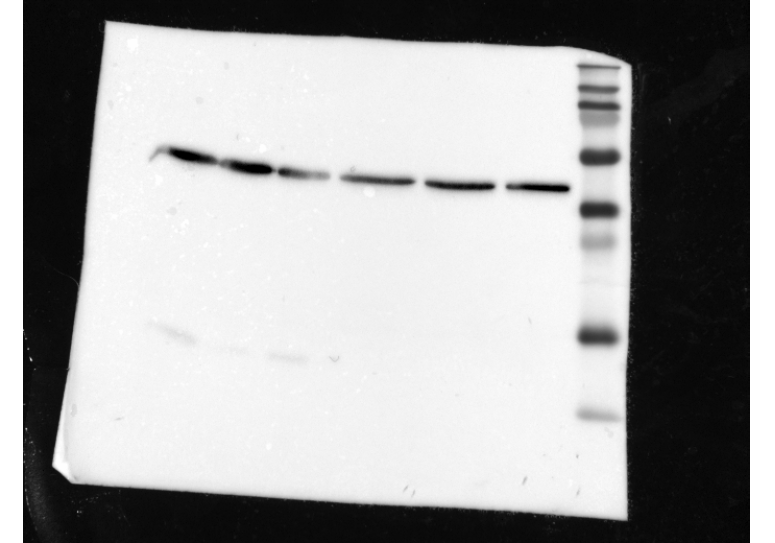


15 D

35 D

55 D

**Figure S2**. In vitro pro-inflammatory stimuli showed no effect on LC3 lipidation in mouse bone marrow-derived *atg7^-/-^* macrophages. Immunoblotting against LC3 in cell lysates of WT and *atg7^-/-^* bone marrow-derived macrophages after 24-h in vitro treatment with (thio) thioglycolate, (cm) conditioned media from thioglycolate-elicited WT macrophages cultured for 24 h and (c) untreated cells. ACTB/β-actin was used as a loading control.
